# Clinical outcomes in carriers of insertional translocation: a retrospective analysis of comprehensive chromosome screening results

**DOI:** 10.1016/j.xfre.2023.11.012

**Published:** 2023-12-02

**Authors:** Zhiqi Zhang, Keli Luo, Senlin Zhang, Dehua Cheng, Liang Hu, Yue-Qiu Tan, Shuoping Zhang, Fei Gong, Pingyuan Xie, Ge Lin

**Affiliations:** a Hospital of Hunan Guangxiu, Hunan Normal University School of Medicine, Hunan, People’s Republic of China; b NHC Key Laboratory of Human Stem and Reproductive Engineering, School of Basic Medical Science, Central South University, Changsha, People’s Republic of China; c Reproductive and Genetic Hospital of CITIC-Xiangya, Hunan, People’s Republic of China; d Clinical Research Center for Reproduction and Genetics, Hunan Province, People’s Republic of China; e National Engineering and Research Center of Human Stem Cells, Changsha, People’s Republic of China

**Keywords:** Insertional translocation, preimplantation genetic testing for structural chromosomal rearrangements (PGT-SR), next-generation sequencing (NGS)

## Abstract

**Objective:**

To evaluate the clinical outcomes in the carriers of insertional translocation (IT).

**Design:**

Retrospective case series.

**Setting:**

University-affiliated reproductive medical center.

**Patients:**

Twenty-three couples with ITs.

**Intervention:**

No direct interventions were involved; however, this study included patients who underwent preimplantation genetic testing for structural chromosomal rearrangements (PGT-SR).

**Main Outcome Measure:**

Outcome of preimplantation genetic testing for structural chromosomal rearrangements and percentage of blastocysts available for transfer.

**Results:**

Among 23 IT carriers, 15 were simple interchromosome ITs (type A), 3 were intrachromosome IT carriers (type B), and 5 were interchromosome IT carriers combined with other translocations (type C). A total of 190 blastocysts from 30 cycles were biopsied, 187 embryos were tested successfully, and only 57 blastocysts (30.5%) from 21 patients were available for transfer (normal or balanced). The unbalanced rearrangement rate of type C was 79.2% (42/53), and the proportion of type A was 50.0% (57/114), which was significantly higher than that of type B (5%, 1/20). In type A, the probability of embryos harboring unbalanced rearrangement in female carriers was 56.0% (51/91), which was higher than that in male carriers (26.1%, 6/23). Furthermore, the haploid autosomal length value of the inserted fragment was correlated linearly with the incidence of abnormal embryos. In type A gametes, most gametes produced by 2:2 separation without crossover, and no 3:1 separation gamete was observed.

**Conclusions:**

The chance of identifying normal or balanced and mosaic blastocysts per mature oocytes in patients with ITs are 16.6% (67/404). Greater IT complexity results in fewer transferable embryos. For simple interchromosome ITs, female carriers and those with higher haploid autosomal length values have a higher risk of producing embryos with unbalanced rearrangement.

Chromosomal insertional translocation (IT) is a rare structural rearrangement involving the transfer of a chromosome fragment to another chromosome (interchromosome IT) or to a different region of the same chromosome (intrachromosome IT). The most common form of chromosomal IT is simple interchromosome IT. This type requires three breaks: one at the donor chromosome and two at the acceptor chromosome. Complex chromosome insertion involves multiple breaks, wherein chromosome fragments may be arranged in a variety of complex ways (1). In 1984, Fryns et al. (2) found only 4 cases of ITs in 40,000 patients who underwent karyotyping. In 2000, Van Hemel and Eussen (3) compared the number of occurrences of Down syndrome–related abnormalities with ITs related with the abnormalities in neonates and concluded that the incidence of ITs in neonates was approximately 1:80,000. Although ITs are rare at the classic cytogenetic level, a study that used array-comparative genomic hybridization identified 40 ITs from approximately 18,000 clinical cases (1:500), which is significantly higher than those in previous studies (4). Generally, IT carriers are phenotypically normal when no gain or loss of the genetic material occurred. However, when the translocation results in the duplication or deletion of fragments or interruption of genes located at the insert site (4), these unbalanced translocations may lead to dysmorphic features, developmental delay (5), or mental deficiency (6).

During meiosis, ITs can produce various gametes with unbalanced chromosomes. In small inserts, the related chromosomes form bivalents and pair independently; after 2:2 and 3:1 segregation patterns, 14 different types (4 for 2:2 and 8 for 3:1) of gametes are produced, and 2 are normal or balanced (Supplemental Fig. 1A and B, available online). However, when the inserted fragment is >1.5% of the haploid autosomal length (HAL), quadrivalents could be formed with a loop, allowing complete pairing of related chromosomes (7). An additional 12 unbalanced gametes formed after 2:2 or 3:1 segregation with crossing over (Supplemental Fig. 1C and D). However, in reference to the segregation pattern, only limited experimental evidence exists. Goldman et al. (8) reported a male IT carrier with an HAL value of 0.18% and examined the chromosome status of 192 sperm. The results showed that the ratio of four possible segregants (duplication, deletion, balanced, and normal) was approximately 1:1:1:1 and no crossover production gametes or 3:1 segregation were identified (8). Melotte et al. (7) reported a case with 1.46% HAL insertion, which produced 10 embryos that contained both normal diploids and unbalanced embryos from 2:2 and 3:1 segregations with crossover. Furthermore, how chromosomes are broken and how chromosome fragments are connected after breakage remain unclear. Because of the risk of producing unbalanced derivative chromosomes during gametogenesis, the fertility of IT carriers is affected (9). Female translocation carriers usually have spontaneous miscarriages risks, whereas male translocation carriers commonly experience reduced sperm quality and quantity (10).

Preimplantation genetic testing for structural rearrangements (PGT-SR) is the most effective way to reduce fertility risk by selecting chromosome-balanced embryos (7). Because of the low incidence of ITs in the population, only few studies have evaluated the genetic risk and clinical outcome of IT. In this study, we classified IT carriers into three types: type A, simple interchromosome IT; type B, intrachromosome IT; and type C, other translocations combined with interchromosome IT. At present, no relevant literature reporting the differences in the genetic risk of the offspring of translocation carriers with different IT types and levels of complexity is available. Therefore, our study will be helpful to assess the genetic risk of IT carriers with different types and levels of complexity.

Here, we focused on the PGT-SR results and pregnancy outcomes. We analyzed the karyotype information of 23 couples, wherein 1 partner was an IT carrier, and the PGT-SR results were based on next-generation sequencing (NGS). We also measured the unbalanced risk of different types of IT carriers and explored the effect of an inserted fragment size on the occurrence of chromosomal abnormalities in embryos. Our results provide useful information for reproductive and genetic counseling for IT carriers.

## Materials and methods

### Patients

Twenty-three couples underwent assisted reproduction with PGT-SR at the Reproductive and Genetic Hospital of CITIC-Xiangya from October 2012 to June 2022. The couples were labeled as cases 1–23. Of the 23 couples, 21 were aged ≤35 years, and 2 were aged >35 years. The mean age was 30 years. Furthermore, 91.3% (21/23) of the couples had a history of abnormal pregnancy including spontaneous miscarriage, induced abortions, and conceiving a child with an abnormal karyotype. Every couple had 1 IT carrier, and 13 female and 10 male carriers participated in the study; 80% (8/10) of the male carriers had low sperm viability or oligospermia (Table 1).

**Table 1:** Clinical characteristics and classification of 23 insertional translocation cases.

Couples	Indication	Female^a^	Male^a^	Female age	IT type^b^
1	Abnormal pregnancy or oligozoospermia	46,XX	46,XY,ins(18;2)(q21;p13p15); chr2: 61742693-74742692	29	A
2	Abnormal pregnancy	46,XX	46,XY,t(4;13)(q28;q13)ins(19;4)(p13;q25)	31	C
3	Abnormal pregnancy	46,XX,ins(7;13)(p13;q22q32); chr13:76620001-95310324	46,XY	32	A
4	Abnormal pregnancy	46,XX	46,XY,ins(7;10)(p15;q22q24); chr10: 90240157-118665825	33	A
5	Abnormal pregnancy or oligozoospermia	46,XX	46,XY,t(4;15;13)(q31;q15;q14)ins(13;4)(q14;q25q31); chr4: 96683278-190190597	24	C
6	Extrauterine pregnancy, recurrent miscarriage	46,XX,ins(1;7)(p22.1;q22q32)	46,XY	30	A
7	Abnormal pregnancy	46,XX,ins(15;5)(q11;q33q22)	46,XY	28	A
8	Abnormal pregnancy	46,XX,der(15)ins(15;2)(q12;q24.2q24.3)	46,XY	34	A
9	Abnormal pregnancy	46,XX	46,XY,ins(1)(q24.1q23.1q25.1)	29	B
10	Abnormal pregnancy	46,XX,ins(8)(q22p12p22); chr8: 5410001-29341854	46,XY	28	B
11	Abnormal pregnancy or low sperm viability	46,XX	46,XY,1qh+,t(7;8)(q21;q21)ins(8;1)(q12;p13p22)	24	C
12	Tubal adhesion or low sperm viability	46,XX	46,XY,ins(14;3)(q32;p24p25)	29	A
13	Abnormal pregnancy	46,XX,t(4;12)(q12;q11),t(12;22)(p13;q11),ins(22;4)(q11;p15p12)	46,XY	28	C
14	Abnormal pregnancy	46,XX,ins(7;12)(q22;q14q21); chr12: 59756695-86756694	46,XY	33	A
15	Abnormal pregnancy	46,XX,ins(13;5)(q32;q11q33); chr5: 61805642-162688727	46,XY	26	A
16	Abnormal pregnancy	46,XX,ins(5;6)(p13;q21q14); chr6: 81678590-103330543	46,XY	32	A
17	Abnormal pregnancy	46,XX,ins(11;6)(p13;q21q25); chr6: 106230544-146139743	46,XY	36	A
18	Abnormal pregnancy or teratozoospermia	46,XX	46,XY,ins(8)(q22p12p22); chr8: 5410001-29341854	29	B
19	Abnormal pregnancy	46,XX	46,XY,ins(5;1)(q33;q32q42); chr1:192909646-221082221	29	A
20	Abnormal pregnancy, insulin resistance	46,XX,ins(3;5)(p23;q21q13); chr5: 77405642-105386128	46,XY	29	A
21	Recurrent miscarriage	46,XX,ins(2;5)(q33;q32q33.3); chr5: 148837074-158388727	46,XY	31	A
22	Abnormal pregnancy	46,XX,inv(9)(p12q13),ins(21;3)(q22;p14p23); chr3:31260001-69270270	46,XY	35	A
23	Abnormal pregnancy or low sperm viability, teratozoospermia	46,XX	46,XY,ins(1;4)(q32.1;q21.3q27)t(1;11)(q32.1;q13.3); chr4: 69889334-110052279	26	C

*Note:* IT = insertional translocation.

a The genomic coordinates of the inserted fragment were added after the karyotype.

b Types of IT carriers: A, simple interchromosome IT; B, intrachromosome IT; and C, interchromosome IT combined with other translocations.

This study was approved by the Institutional Review Board (number, LL-SC-2023-013) and conducted in accordance with the Helsinki Declaration ethical principles. Counseling was provided to all couples before signing informed consent forms.

### Cytogenetic Analysis

The peripheral blood lymphocytes collected from all couples were cultured using the standard G-banding technique. A total of 20–30 GTG lymphocytes in the middle stage were detected with complete analysis of 7–8 karyotypes in each patient. The karyotypes in this report were described according to the International System for Human Cytogenomic Nomenclature 2016 guidelines.

To evaluate the occurrence of ITs on different chromosomes, ITs from 1972 to 2022 were searched in the National Center for Biotechnology Information PubMed database, and only one case in a single family was included. Studies with ITs on the sex chromosome and without complete translocation karyotype records were excluded.

### NGS-Based PGT-SR

Next-generation sequencing–based PGT-SR was performed as described previously (11, 12). Patients were treated with personalized ovulation induction regimens according to their situations. Four to six hours after oocyte retrieval, intracytoplasmic sperm injection was used to fertilize all mature oocytes. All embryos after fertilization were cultured in G1 and G2 media (Vitrolife, Gothenburg, Sweden) at 37°C using an incubator (Cook, Bloomington, IN). The embryos were carefully cultured to blastocyst under the following conditions: 6% carbon dioxide; 5% oxygen; and 89% nitrogen. Blastocyst trophoblast biopsy was performed on the morning of day 5 or 6 after fertilization, and approximately five trophoblast cells were obtained using ZILOS-tk Laser (Hamilton Thorne, Beverly, MA) (13). Next, the whole genome of the biopsied cells was amplified using the REPLI-g Single Cell Kit (Qiagen, Hilden, Germany) or PicoPLEX Whole Genome Amplification Kit (Rubicon Genomics, Ann Arbor, MI), following the manufacturer’s instructions. As previously described, NGS and comprehensive chromosome screening were performed to select balanced embryos (14). Embryos were characterized as normal or balanced, aneuploid, or mosaic (30%–70%), as described previously (15). Normal or balanced and mosaic embryos with a low degree (30%–50%) after screening were transferred to the uterine cavity in a frozen embryo transfer cycle.

### Statistical Analysis

The chromosome relative length (16) and calculation method (occurrence of breakpoints on chromosome(s) length [megabase]) (1) reported previously were used to calculate the occurrence of ITs on each chromosome that excluded the effect of chromosome size, which was different for each autosome. The HAL values were calculated using the following formula: length (millimeter) of the inserted fragment divided by the length (millimeter) normal of the donor chromosome, as described previously (16). The length (millimeter) of the inserted fragment was obtained from Genome Reference Consortium Human GRCh38.p14. Linear fits between the HAL values and disequilibrium rates were performed using the ORGIN software (17). The Cochrane and Mantel-Haenszel χ^2^ test was used to explore the interaction effect of the carrier’s sex on the likelihood of each single embryo to have an unbalanced rearrangement. Linear correlation analysis was used to evaluate the relationship between the HAL value and proportion of unbalanced rearrangement. A *P* value of <.05 was considered significant.

## Results

### Distribution of IT

**Figure 1:**
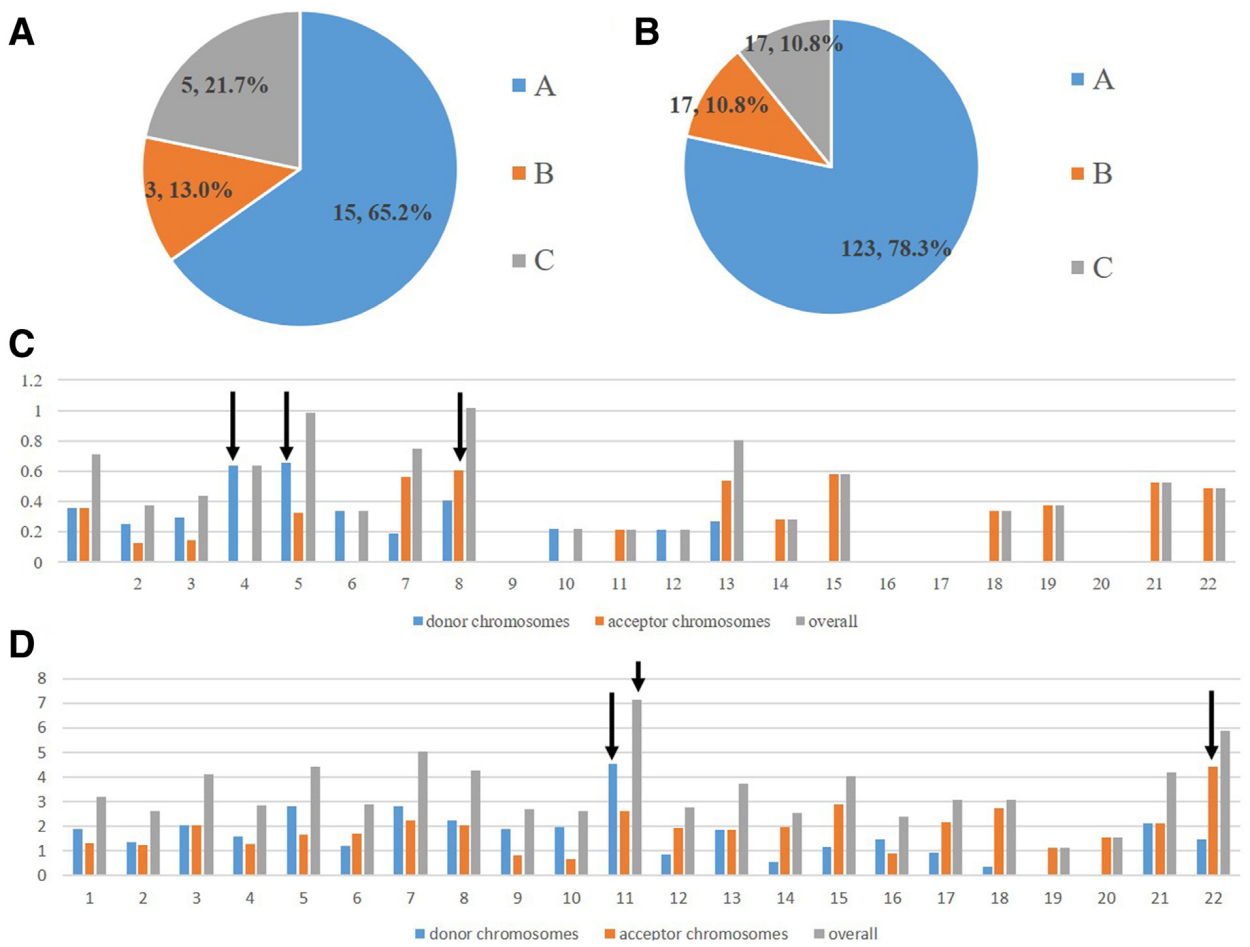
The proportion of insertional translocation (IT) types and incidence of ITs on different chromosomes. (**A**) The proportion of three different types of IT carriers in our study. (**B**) The proportion of three different types of IT carriers in the literature. (**C**) The relative incidence of ITs on different chromosomes in our study. (**D**) The relative incidence of ITs on different chromosomes in the literature. The arrows indicate the chromosomes with a higher incidence of translocation.

The karyotypes of the 23 couples on the basis of the G-banding analysis are shown in Table 1. Of the 23 couples, 11 female and 4 male partners were type A IT carriers, 1 female and 2 male partners were type B IT carriers, and 1 female and 4 male partners were type C IT carriers. Type A, simple interchromosome, ITs accounted for the highest proportion (60.9%) of carriers (Fig. 1A). We also reviewed 157 cases from 142 studies that reported ITs between 1972 and 2022 (Supplemental Table 1), among which simple interchromosome ITs accounted for the highest proportion (78.3%) (Fig. 1B).

To understand the occurrence of ITs on different chromosomes, we calculated the incidence of breakpoints on donor and recipient chromosomes of 23 translocation carriers. In this study, ITs were more common on chromosomes 1–8, and chromosomes 4 and 5 and chromosome 8 accounted for the highest proportion of donor and recipient chromosomes, respectively (Fig. 1C). However, combining our data with those in the literature, chromosome 11 had the highest incidence of translocation and proportion of donor chromosome, whereas chromosome 22 had the highest proportion of recipient chromosome (Fig. 1D).

### Clinical Characteristics

A total of 30 PGT-SR cycles were performed, among which 5 were performed in case 5, 3 in case 21, 2 in case 15, and 1 in the other cases. More than 5 oocytes were obtained in each cycle, and a total of 495 oocytes were obtained, of which 404 (81.6%) were available for fertilization. A total of 378 (76.4%) were fertilized. A total of 190 blastocysts were obtained for biopsy, of which 187 were tested successfully. A total of 57 (30.5%) and 10 (5.3%) blastocysts were characterized as normal or balanced and mosaic embryos, respectively. A total of 26 blastocysts from 21 patients were transferred, and 17 pregnancies and 15 live birth deliveries (57.7%) were achieved (Supplemental Table 2).

### Analysis of Unbalanced Rearrangement

Among the 187 blastocysts tested using PGT-SR, 83 (44.4%), 17 (9.1%), and 20 (12.7%) embryos carried translocation-related abnormalities, both translocation-related and emerging abnormalities, and new emerging abnormalities, respectively. To evaluate the effects of different IT types and sexes on the frequency of unbalanced rearrangement blastocysts, we divided the embryos into two categories: embryos with and without unbalanced rearrangement originating from parental IT (Table 2). The results indicated that type C (79.25%) had the highest unbalanced rearrangement rate, followed by types A (50%) and B (5%) (Table 2). Furthermore, in type A, the unbalanced rearrangement ratio in female carriers (56.04%) was significantly higher than in male carriers (Table 2). Considering the previous studies suggesting that the HAL values are closely related to IT gametogenesis, we selected the cases in type A with >4 embryos and then analyzed the effect of the HAL value on unbalanced rearrangement. The results exhibited a moderate correlation between the HAL value and rate of abnormalities in embryos, with no significant difference (r = 0.606, *P* = .084) (Supplemental Fig. 2).

**Table 2:** Occurrence of unbalanced rearrangements in different types and sexes of insertional translocation carriers.

Groups	Unbalanced (%)	Balanced (%)	*P* value	OR (95% CI)
Types				
A	57 (50)	57	<.001	19.000 (2.460–146.725)
B	1	19		Reference
C	42	11	<.001	72.545 (8.728–602.951)
Carrier sex of type A				
Male	6	17	.01	0.277(0.100–0.767)
Female	51	40		Reference

*Notes:* CI = confidence interval; OR = odds ratio.

### Segregation Pattern Analysis

**Figure 2:**
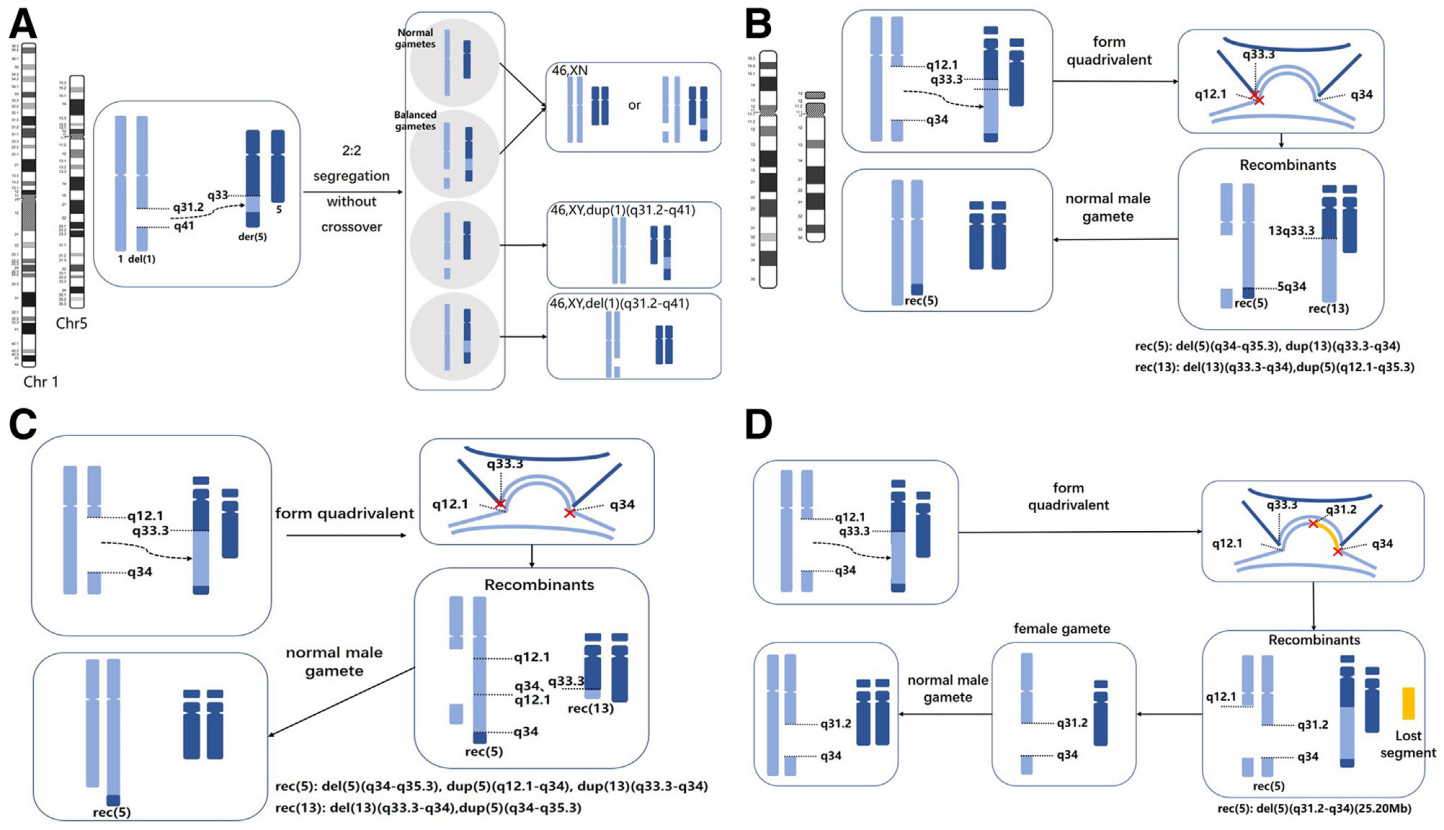
The pattern of gametogenesis in insertional translocation identified in blastocysts. (**A**) Four types of gametes produced after 2:2 separation without crossover. (**B–D**) Three types of gametes produced after 2:2 separation with crossover. (**B**) The first type of break occurred at the insert site of the recipient chromosome (13q33.3) and near the centromeric site of the donor chromosome (5q12.1). (**C**) The second type of break occurred at the insert site of the recipient chromosome (13q33.3) and at the far centromeres of the donor chromosome (5q34). (**D**) The third type of breakage occurred in the donor chromosome twice (5q31.2 and 5q34), resulting in the loss of a small fragment. The detailed PGT-SR results are shown in Supplemental Table 2 and Supplemental Figure 3.

To analyze the potential segregation pattern of ITs in blastocysts, we analyzed the segregation pattern of type A. Most of the cases (cases 1, 3, 4, 8, 12, 14, 16, 17, 19, 20, 21, and 22) had only four types of embryos: normal karyotype; balanced karyotype; absence of the translocation fragment; and duplication of the translocation fragment, which had a segregation pattern of 2:2 without crossover occurring (Fig. 2A).

Interestingly, case 15 had a large inserted fragment (3.4% HAL), which produced an extremely complex karyotype. After sorting, seven different segregation types were identified (Supplemental Table 3 and Supplemental Fig. 3). The first four types of gametes were produced after a segregation pattern of 2:2 without crossover as aforementioned cases.

The last three types of embryos were all generated after crossover. In detail, the first type (Fig. 2B) of break occurred at the insert site of the recipient chromosome (13q33.3) and near the centromeric site of the donor chromosome (5q12.1). The second type (Fig. 2C) of break occurred at the insert site of the recipient chromosome (13q33.3) and at the far centromeres of the donor chromosome (5q34). The third type of breakage occurred in the donor chromosome twice (5q31.1 and 5q34), resulting in the loss of a small fragment, forming the recombinant chromosome rec(5): del(5)(q31.2-q34)(25.20Mb) (Fig. 2D).

## Discussion

Meiotic segregation analyses of ITs in blastocysts are rarely explored because of their low incidence. In this study, we analyzed the PGT outcomes and blastocyst separation patterns in 23 couples, in which 1 partner was an IT carrier. The results showed that 56.5% of the couples achieved good pregnancy outcomes on the basis of PGT-SR. At the same time, we found that the proportion of chromosomal abnormalities was high (>50%). Therefore, natural pregnancy in IT carriers is not encouraged, and those with natural conception should undergo prenatal diagnosis.

The frequency of ITs was different across the 23 pairs of chromosomes. In the study by Dong et al. (1), chromosome 2 had a significantly higher incidence both in the overall insertion and as a donor chromosome. In our study, chromosomes 5 and 8 had the highest frequency of translocations, whereas in the literature, it was chromosome 11. Because ITs are rare chromosomal rearrangements, more cases are needed to discuss the frequency of ITs occurring on different chromosomes.

Our study showed that the risk of unbalanced rearrangement varies between different IT types and sexes. The results indicated that the more chromosomes involved and the more complex the translocation, the higher the risk of inheritance. Xanthopoulou et al. (18) found that among intrachromosome IT carriers, a significantly lower proportion of translocation-related abnormal embryos were produced in female carriers than in male carriers. However, the opposite was true in simple interchromosome IT carriers in our study. Xanthopoulou et al. (18) analyzed only 1 male and 1 female intrachromosome IT carrier, whereas we analyzed 11 female and 3 male interchromosome IT carriers. The higher risk of unbalanced rearrangements in female carriers is also consistent with the results in reciprocal translocation (19) and Robertsonian translocation carriers (20). Furthermore, the sperm carrying unbalanced rearrangements may fail to mature during spermatogenesis (21).

A moderate linear increase in the risk of unbalanced rearrangement with the size of the translocated fragment was observed. However, because of the limited number of cases and embryos, no significant differences were observed. Previous studies on inversion carriers have also suggested that an inverted segment size ratio was a significant risk factor for unbalanced rearrangement (12), which is similar to our results. More cases are needed to further evaluate the relationship between the translocation fragment size of ITs and unbalanced rearrangement.

In the meiotic behavior of ITs, previous studies have suggested that, in theory, at least 26 possible segregants can result from the meiotic process. However, little experimental evidence exists, and it is unclear where the breaks occur and how the new broken segments combine when forming a recombinant chromosome. Our results indicated that when the inserted fragment is relatively small, meiosis could proceed in the usual fashion, with the absence of looping out and no quadrivalent formation. Only four types of gametes are generated: normal; balanced; translocation fragment duplication; and deletion. However, when the inserted fragment was large (>1.5% HAL), a quadrivalent was formed, thereby enabling recombination within the insertional segments. We identified 3 distinct patterns of break occurrence by analyzing the PGT-SR results of 19 blastocysts in IT carriers with a large inserted fragment (46,XX,ins(5, 13)(q32;q11q33)). To note, no 3:1 separation pattern mentioned in the literature was identified in the present study. From this, we speculated that the occurrence of 3:1 segregation pattern in ITs was rare, which was also rare in blastocysts from Robertsonian translocation carriers (20). Overall, our results may help in exploring the gametogenesis mechanism of IT. However, it should be noted that the results were based on the blastocysts, which cannot fully simulate the gametogenesis of IT carriers. Future studies in sperm may provide a clearer picture of the rearrangement pattern of the ITs.

Although our study provides some suggestions for genetic counseling for carriers of different translocation types and sexes, it still has limitations. First, our sample size was small because of the low incidence of ITs in the population. The patients were all carriers who appeared to have a normal phenotype and pursued assisted reproduction in our hospital, which cannot represent the total IT population and may bring some deviation. Second, our segregation pattern analysis was based on blastocysts, which may not reflect the actual segregation pattern in gametes.

In conclusion, the meiotic segregation analyses of different IT types in blastocysts suggested greater IT complexity results in fewer transferable embryos. Interchromosome ITs combined with other translocations have a higher risk. In addition, in common interchromosome ITs, female carriers and those with higher HAL values have a higher risk of producing embryos with unbalanced rearrangement. Our results may provide more appropriate information for couples with ITs. Further studies are required to explore the formation mechanism and segregation pattern of ITs.

## Declaration of Interests

Z.Z. has nothing to disclose. K.L. has nothing to disclose. Se.Z. has nothing to disclose. D.C. has nothing to disclose. L.H. has nothing to disclose. Y.-Q.T. has nothing to disclose. Sh.Z. has nothing to disclose. F.G. has nothing to disclose. P.X. has nothing to disclose. G.L. has nothing to disclose.

## Supplementary data

Supplementary Results
