# Supplementary material for: Clinical outcomes in carriers of insertional translocation: a retrospective analysis of comprehensive chromosome screening results
Source: F S Rep. 2023 Dec 2;5(1):55–62. doi: 10.1016/j.xfre.2023.11.012 (PMC10958698; doi:10.1016/j.xfre.2023.11.012)
Supplement: Supplementary Results [file mmc1.docx]

| **Supplementary Table 1** | | | |
| --- | --- | --- | --- |
| Serial number | Karyotype | ITs Types | Cite |
| 1 | 46,XX,ins(4;1) | A | (1) |
| 2 | 46,XX,ins(3;7)(q27;q22q32) | A | (2) |
| 3 | ins(7;3)(q31;p21;p26) | A | (3) |
| 4 | 46,XY,ins(12;2) | A | (4) |
| 5 | 46,XX, ins (17;5) (q2l;pl4→pter) | A | (5) |
| 6 | 46,XX or XY, ins(11;18)(p15;q11q21) | A | (6) |
| 7 | 46, XX, inv ins (4; 2) (q24; q22q34) | A | (7) |
| 8 | 46XX or XY, ins(1)(p32q25q31) | B | (8) |
| 9 | ins(3;5) (q27;q13q15) | A | (9) |
| 10 | ins(15;10) (q15;q24q26) | A | (10) |
| 11 | 46,XX,ins(7)(p15p21;q22) | B | (11) |
| 12 | 46,XX,ins(12;13) (p11.2;q22.1q12.5) | A | (12) |
| 13 | 46,XX,ins(18;4)(q22;q12q13),t(2;4)(p24;q13). | C | (13) |
| 14 | 46,XX,ins(7;13)(q22;q32q34) | A | (14) |
| 15 | 46,XX, or XY,der(7)ins(13;7) (q32;q32q34) | A | (15) |
| 16 | 46,XX,t(9;22)(q34;q11),ins(11)(q23p15p12) | C | (16) |
| 17 | 46,XX or XY, dir ins(l1;3) (q22.l;q22.lq24) | A | (17) |
| 18 | ins(3;13)(p12;q13.1-q14.5) | A | (18) |
| 19 | 46, XX, ins(22;9) (q11;q22 to 34) | A | (19) |
| 20 | ins(10;5) (q22;q13;q22) | A | (20) |
| 21 | ins(2)(p13;q31q32) | B | (21) |
| 22 | ins(19;11)( p13;q13q23) | A | (22) |
| 23 | 46,XX,ins(15;10)(q22;q22q24) | A | (23) |
| 24 | ins(4;3)(p?;p21p22) | A | (24) |
| 25 | ins(2;14)(p11;q11q13) | A | (25) |
| 26 | ins(5;3)(q23;p22p26) | A | (26) |
| 27 | 46,XX,inv ins(5;10)( q13;q24.2q25.3) | A | (27) |
| 28 | 46,XY,inv ins(12;21)(p11;p12q11) | A | (28) |
| 29 | 46,XX,dir ins(11;22)(q13;q11q13) | A | (29) |
| 30 | ins(4;11)(q21;q23) | A | (30) |
| 31 | 46, XX, dir ins (1:5) (p31;q34q22) | A | (31) |
| 32 | ins (5) (p15q12q12) | B | (32) |
| 33 | ins(10;11) (p11; q23q24) | A | (33) |
| 34 | inv ins (5;8)(p13;q12q21.2) | A | (34) |
| 35 | 46,XY,ins(6;11)(p22;q21q25) | A | (35) |
| 36 | 46,X,t(X;18)(pll.2;q11.2)dir ins(15;11)(q24;q22.2q25) | C | (36) |
| 37 | 46,XY, ins(11)(p14.2q23.3q24.2) | B | (37) |
| 38 | 46,XX,inv ins(3;5)(p13;p15p13) | A | (38) |
| 39 | dir ins(14;6)(q23;p23p25) | A | (39) |
| 40 | 46,XY, inv ins(1,3)(q32;p3pter), | A | (40) |
| 41 | 46,X,t(X;18)(p11.2;q11.2),dir ins(15;11)(q24;q22.2q25) | C | (40) |
| 42 | ins(14;11)(q23;p12p14) | A | (41) |
| 43 | 46,XY,dir ins(20;5)(q11.2;q11.2q13.1) | A | (42) |
| 44 | 46,XY,ins(14;16)(q23;q13q22) | A | (43) |
| 45 | 46,XY, t(2;13;16) (q11;q12;p11), ins(6;7)(p21.3;q21.3q35) | C | (44) |
| 46 | 46,XX,dir ins(7;1)(p15.3;q12q21.3) | A | (45) |
| 47 | 46,XY,ins(X) (p11.4q13.3q21.2) | B | (46) |
| 48 | ins (3;10) (q13.2;p14p13) | A | (47) |
| 49 | ins(8)(q13;q23q24.2) | B | (48) |
| 50 | dir ins (13;11)(q14.1; p11.12p12) | A | (49) |
| 51 | ins(3;7)(q21;q22q34) | A | (50) |
| 52 | ins(10;11)(p11;q23q24) | A | (51) |
| 53 | 46,XX,ins(7;8) (q36;p11.2p23) | A | (52) |
| 54 | ins(9;3)(p23;p11-?) | A | (53) |
| 55 | 46,XX,dir ins (18;3)(p11.1;q13.2q25) | A | (54) |
| 56 | ins(8;3)(q12;p21.3p14.1) | A | (55) |
| 57 | ins(12;10)(q15;q21.2q22.1) | A | (56) |
| 58 | ins(17;15)(p13;q22.3q23 or 24) | A | (57) |
| 59 | ins(12;16)(q13;p11.2p13) | A | (58) |
| 60 | der(13) ins(13;5)(q34;q12q13.3) | A | (59) |
| 61 | 46,XY,dir ins(2;4)(p24;p15.3p13) | A | (60) |
| 62 | ins(9;7)(p21;q21433?) | A | (61) |
| 63 | ins(17)(p11.2q11.2q21.3) | B | (62) |
| 64 | ins(6;11)(q27;q13q23) | A | (63) |
| 65 | ins(X;1)(q21;q12q21) | A | (64) |
| 66 | 46,XY der(6)ins(6;7)(q16.2;q212q221) | A | (65) |
| 67 | ins(6;1)(q25;p13.3p22.1) | A | (66) |
| 68 | ins(3;1)(q25q27; q13-14 to q34-35) | A | (67) |
| 69 | t(1;9;22)(q25;q34;q11.2)ins(17;22)(p12-13;q11.2q11.2) | C | (68) |
| 70 | 46,XY,ins(17;2)( q25; q35q37.1) | A | (69) |
| 71 | 46,XY,ins(1;16)(q42;p13.1p13.3) | A | (70) |
| 72 | dir ins (5;11)(p14;q14q24) | A | (71) |
| 73 | ins(11;13)(q14q122)(q21.32q31.2) | A | (71) |
| 74 | dir ins (18;5)(q21.3;p13.1p14) | A | (71) |
| 75 | ins (18;12)(p11.3;q13q15) | A | (71) |
| 76 | inv ins(5;10)(q15;q26.3q25.2) | A | (71) |
| 77 | 46,XX,der(2)ins(2;11)(q32.2;q22.3?q22.3)t(2;16)(q32.2;q13) | C | (72) |
| 78 | 46,XY,t(7;9)(q22;p24),ins(8;7)(q21.2;q22q32) | C | (73) |
| 79 | ins(22;9)(q11;q34q21) | A | (74) |
| 80 | t(7;18)(q22;p11.3) ins(20;Y)(q12;q11q13) | C | (75) |
| 81 | ins(18;13)( q23; q12.3q21.3) | A | (76) |
| 82 | 46,XX, ins (2;5) (q14.1;q14.1q23.2) | A | (77) |
| 83 | ins(15;17)(q22;q12q21) | A | (78) |
| 84 | ins(1;7)(p32;q32q35) | A | (79) |
| 85 | ins(12;15)(p13;q22q26) | A | (80) |
| 86 | 46,XX,ins(12;9)(q24.1;p22p24) | A | (81) |
| 87 | del(20)(q11q13)ins(20;21)(q11;q11q22) | A | (82) |
| 88 | ins (16;6)(p12;p21.2p23) | A | (83) |
| 89 | 46,XY,t(3;9)(p11;p23), ins(8;9)(q23;p23) | C | (84) |
| 90 | 46,XY, der(20)ins(20;5)(p13;q31.1q35.1) | A | (85) |
| 91 | 46,XY,ins(12;3)(p13;q?21q?22) | A | (86) |
| 92 | 46,X,ins dup(Y)( p11.23;q12 q11.1;p11.23) | B | (87) |
| 93 | 46,XX,der(13)ins(11;13)(p13;q1314.1q14.321.1) | A | (88) |
| 94 | ins(22;12)(q12;q13q14) | A | (89) |
| 95 | ins(4;11)(q21;q13q23) | A | (90) |
| 96 | 45,X,-Y,ins(21;8)(q22;q22q22) | A | (91) |
| 97 | t(15;17), ins(17;15)(q21;q14q22) | C | (92) |
| 98 | 46,XY,ins(1;11)(q22-q23;q13q23),t(11;22)(q13;q11-q12) | C | (93) |
| 99 | 46,XY,der(2)inv(2)(q13q21)inv(2)(q21q24.2)ins(2)(q24.2q33q35) | C | (94) |
| 100 | ins(12;6)( p13;p22.522.4) | A | (95) |
| 101 | 46, XX, der(1),t(1;11)(p13.3;q14.3), der(7),t(1;7)(p21;q31.1),ins(7;8)(q31.1;  p11.2p11.1), der(8),t(7;8)(q31.2;p11.1), der(11),t(8;11)(p11.2;q14.2) | C | (96) |
| 102 | ins(11;X) (q23;q28q12) | A | (97) |
| 103 | ins(3;5)(q25.3;q22.1q31.3) | A | (98) |
| 104 | 46,XX,ins(14;2)(q21;q31q35) | A | (99) |
| 105 | 46,XX,ins(15;10)(q22;q22q24) | A | (100) |
| 106 | t(21;22), ins(22;21)(q12;q21q22) | C | (101) |
| 107 | 46,XY,.ish der(22),ins(22;16)(p13;p13.3p13.3) | A | (102) |
| 108 | ins(4;X)(q31-32;p11p22) | A | (103) |
| 109 | ins(6;7)(p25;q33q34) | A | (104) |
| 110 | ins(4;11)(q21;q23q23) | A | (105) |
| 111 | ins(15;14)(q22;q13q32) | A | (106) |
| 112 | 46,XY,der(7)ins(5; 7) (q35; p13p14)pat | A | (107) |
| 113 | ins(15;17)(q22;q21.1q21.3) | A | (108) |
| 114 | 46,XY, t(3;11)(q27; q13), ins(11;3)(q13;p26p13) | C | (109) |
| 115 | inv ins(2;4)(p24;q28.3q31.22) | A | (110) |
| 116 | ins(4;11)(p14;q24q25) | A | (111) |
| 117 | ins(2;6)(q37.2;p21.1p12.3) | A | (112) |
| 118 | ins(8;21)(q22;q22q22) | A | (113) |
| 119 | ins(3)(q26.2;q21q26.2) | B | (114) |
| 120 | ins(11;1)(q23;q21q31) | A | (115) |
| 121 | ins(7;6)(p15;q16.1q21) | A | (116) |
| 122 | ins(21;8)(q22;q22q22) | A | (117) |
| 123 | 46,XY,ins(3;2)(p23;q23q14.2),t(6;14)(p12.2;q13) | C | (118) |
| 124 | 46, XX, inv ins (3; 8) (q25.3; p23.1p11.2) | A | (119) |
| 125 | 46,XX,der(19)ins(19;9)(q13.4;q34.3q34.3) | A | (120) |
| 126 | 46, XX, ins(1; 7) (p32; q32q35) | A | (121) |
| 127 | ins(8; 4) (p11.12; q13.2q22.1) | A | (122) |
| 128 | 46,XX,ins(1)(q42.11q42.3) | B | (123) |
| 129 | 46,XY,ins(8)(p23.1p23.1) | B | (124) |
| 130 | 46,XY.ish ins(22;9)(q11.2;q34q34) | A | (125) |
| 131 | ins(21;4)(q21;q13.1q13.3) | A | (126) |
| 132 | 46,XX,ins(13;8)(q33;q23q24) | A | (127) |
| 133 | der(9)ins(9;4)(q34.3;q26q35.2) | A | (128) |
| 134 | ins(6;12)(p22.3p13) | A | (129) |
| 135 | ins(9) (q21.32q31.1) | B | (130) |
| 136 | 46, XY, inv ins (18,7) (q22.1; q36.2q21.11) | A | (131) |
| 137 | ins(18;5)(q21.1;q31.2q35.1) | A | (132) |
| 138 | ins(X;11)(q28;q23q23) | A | (133) |
| 139 | 47, XY, add(3)(q29), -7, ins(17;15)(q12;q14q22),+21,+mar | A | (134) |
| 140 | ins (22;9) (q11;q21q34) | A | (135) |
| 141 | ins(11;X)(q23;q28q12) | A | (136) |
| 142 | 46,XX,ins(22;1)(q13;p13p31) | A | (137) |
| 143 | 46,XX,ins(14;1)(q23.1;q43q44) | A | (138) |
| 144 | 46,XY,ins(7;1)(p11.2;q31.3q41) | A | (139) |
| 145 | 46,XX,der(7)ins(7)(q31.32;p14.1p15.1) | B | (140) |
| 146 | 46,XY,der(3)ins(3;1)(q23;p21.1p21.3) | A | (140) |
| 147 | 46,XY,der(5)ins(5;1)(q14.2;q32.1q32.2) | A | (140) |
| 148 | 46,XY,der(6)ins(6;2)(q16.2;q23.3q31.1) | A | (140) |
| 149 | 46,XY,der(2)ins(2;11)(p21?;q22.1q24.2) | A | (140) |
| 150 | 46,XX,der(3)ins(3)(q27;p25.3p21.31) | B | (140) |
| 151 | 46,XX,der(16)ins(16)(p13.3;q22.1q23.1) | B | (140) |
| 152 | 46,XY,der(16)ins(16;2)(q23.1;p22.2p21) | A | (140) |
| 153 | 46,XY,der(X)ins(X)(q2?;p11.21p11.22) | B | (140) |
| 154 | 46,XY,der(16)ins(6;9)(p24.1;q33.2q34.3) | A | (140) |
| 155 | 46,XX,der(13)ins(13;10)(q22;p12.31p11.22) | A | (140) |
| 156 | ins(14;22)(q11.2;q11.2q11.2) | A | (141) |
| 157 | ins(6;11)(q27;q23q23) | A | (142) |

**Supplementary Table 2: Detail PGT results of 30 PGT-SR cycles.**

| **Couples** | **ITs types#** | **Numbers of retrieved oocytes** | **Numbers of Oocytes available for fertilization** | **Number of fertilized oocyte** | **Number of biopsied embryos** | **Normal/balanced** | **Mosaicism** | **Number of transferable cycle** | **pregnancies** | **live birth** |
| --- | --- | --- | --- | --- | --- | --- | --- | --- | --- | --- |
| **1** | A | 18 | 18 | 17 | 6 | 5 | 0 | 2 | yes | yes |
| **2** | C | 6 | 6 | 4 | 2 | 0 | 0 |  |  |  |
| **3** | A | 12 | 11 | 10 | 4 | 2 | 0 | 1 | yes | yes |
| **4** | A | 13 | 11 | 11 | 7 | 3 | 0 | 1 | yes | yes |
| **5-1** | C | 8 | 5 | 4 | 4 | 1 | 0 | 1 | yes | no |
| **5-2** | C | 18 | 15 | 14 | 1 | 0 | 0 |  |  |  |
| **5-3** | C | 36 | 23 | 19 | 5 | 2 | 0 | 2 |  |  |
| **5-4** | C | 21 | 19 | 19 | 11 | 0 | 1 | 1 | yes | yes |
| **5-5** | C | 9 | 9 | 7 | 7 | 0 | 0 |  |  |  |
| **6** | A | 5 | 1 | 1 | 1 | 1 | 0 | 1 | yes | yes |
| **7** | A | 10 | 8 | 7 | 1 | 1 | 0 | 1 |  |  |
| **8** | A | 9 | 7 | 7 | 6 | 1 | 1 | 1 | yes | yes |
| **9** | B | 23 | 21 | 19 | 10 | 7 | 1 | 1 | yes | yes |
| **10** | B | 28 | 19 | 19 | 9 | 5 | 2 | 1 | yes | yes |
| **11** | C | 13 | 12 | 11 | 5 | 1 | 0 | 1 | yes | yes |
| **12** | A | 8 | 7 | 7 | 4 | 0 | 0 |  |  |  |
| **13** | C | 12 | 8 | 6 | 2 | 1 | 0 | 1 | yes | yes |
| **14** | A | 24 | 18 | 16 | 7 | 2 | 0 | 1 |  |  |
| **15-1** | A | 16 | 11 | 11 | 6 | 0 | 1 |  |  |  |
| **15-2** | A | 23 | 17 | 17 | 13 | 3 | 0 | 1 | yes | yes |
| **16** | A | 24 | 24 | 24 | 15 | 5 | 1 | 1 | yes | yes |
| **17** | A | 14 | 10 | 10 | 6 | 2 | 0 | 1 | yes | yes |
| **18** | B | 29 | 24 | 24 | 1 | 0 | 1 |  |  |  |
| **19** | A | 14 | 11 | 10 | 7 | 4 | 0 | 1 | yes | no |
| **20** | A | 18 | 12 | 10 | 6 | 3 | 0 | 1 | yes | yes |
| **21-1** | A | 13 | 12 | 12 | 3 | 0 | 0 |  |  |  |
| **21-2** | A | 20 | 19 | 17 | 12 | 3 | 0 | 2 |  |  |
| **21-3** | A | 25 | 21 | 20 | 11 | 2 | 0 | 1 |  |  |
| **21-4** | A | 5 | 4 | 4 | 3 | 1 | 0 | 1 |  |  |
| **23** | C | 21 | 21 | 21 | 12 | 2 | 2 | 1 | yes | yes |
| **Total** |  | **495** | **404** | **378** | **187** | **57** | **10** | **26** | **17** | **15** |

**#A:** simple inter-chromosome Its; B: intra-chromosome Its; C: inter-chromosome ITs combined with other translocations

| **Supplementary Table 3 the detailed biopsy results of 15 embryos in case 15** | | | | |
| --- | --- | --- | --- | --- |
| Cycle | Embryo number | NGS result | types | segregation types |
| 15-1 | 1 | 46,XN,del(5)(q34-q35.3)(18.40Mb),dup(13)(q33.3-q34)(7.9Mb) | Translocation related anomalies | 2:2 with crossover and the first break mode* |
| 15-1 | 2 | 46,XN,del(5)(q12.1-q34)(100.18Mb),del(20)(p13-p12.1)(17.28Mb) | Translocation related anomalies | 2:2 with crossover and the third break mode* |
| 15-1 | 3 | 46,XN,del(5)(q12.1-q34)(100.48Mb) | Translocation related anomalies | 2:2 without crossover |
| 15-1 | 4 | 46,XN,dup(5)(q12.1-q34)(100.48Mb),del(5)(q34-q35.3)(18.11Mb),dup(13)(q33.3-q34)(7.73Mb) | Translocation related anomalies | 2:2 with crossover and the second break mode* |
| 15-1 | 5 | 45,XX,-12 | Other anomalies |  |
| 15-1 | 6 | 46,XN,+12(mosaic,35%),-21(mosaic,35%) | Other anomalies |  |
| 15-2 | 7 | 46,XN | Normal/Balanced | 2:2 without crossover |
| 15-2 | 8 | 45,XY,-13 | Translocation related anomalies | 3:1 without crossover |
| 15-2 | 9 | 46,XN,dup(5)(q12.1-q34)(100.88Mb) | Translocation related anomalies | 2:2 without crossover |
| 15-2 | 10 | 46,XN,del(5)(q34-q35.3)(18.40Mb),dup(13)(q33.3-q34)(7.90Mb) | Translocation related anomalies | 2:2 with crossover and the first break mode* |
| 15-2 | 11 | 46,XN,del(5)(q12.1-q34)(100.48Mb) | Translocation related anomalies | 2:2 without crossover |
| 15-2 | 12 | 45,XO,dup(5)(q12.1-q34)(100.88Mb) | Translocation related anomalies | 2:2 without crossover |
| 15-2 | 13 | 46,XN,dup(5)(q12.1-q35.3)(118.88Mb),dup(13)(q12.11-q33.3)(87.89Mb) | Translocation related anomalies | 2:2 with crossover and the first break mode* |
| 15-2 | 14 | 46,XN | Normal/Balanced | 2:2 without crossover |

*The first break mode: break occurred at the insert site of the recipient chromosome and near the centromeric site of the donor chromosome.

The second break mode: break occurred at the insert site of the recipient chromosome and at the far centromeres of the donor chromosome.

The third break mode: break occurred in the donor chromosome twice, resulting in the loss of a small fragment.

**
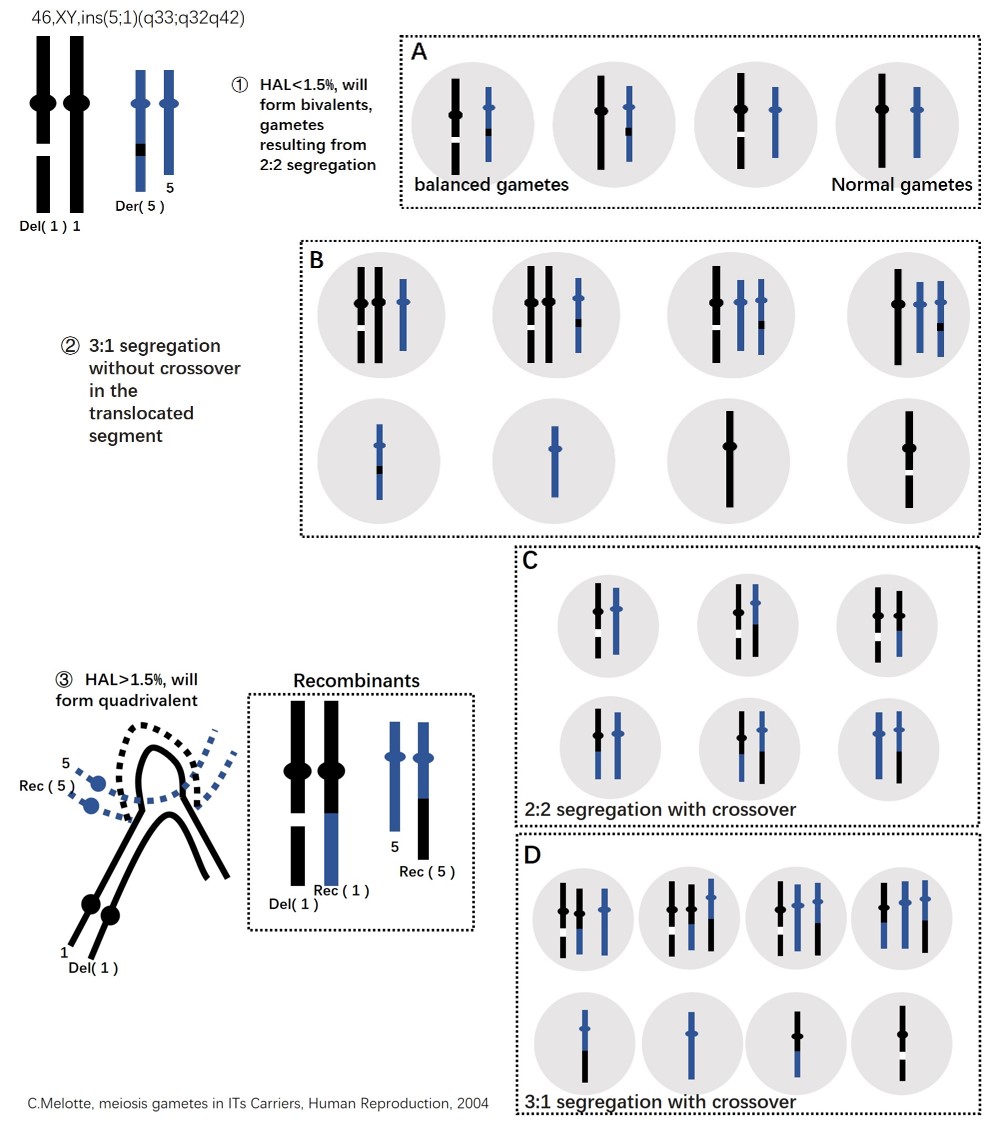
**

Supplementary Figure 1: Pattern diagram of the production of 26 gametes by insertion translocation. A: 2:2 separation without crossover; B: 3:1 separation without crossover; C: 2:2 separation with crossover; D: 3:1 separation with crossover.


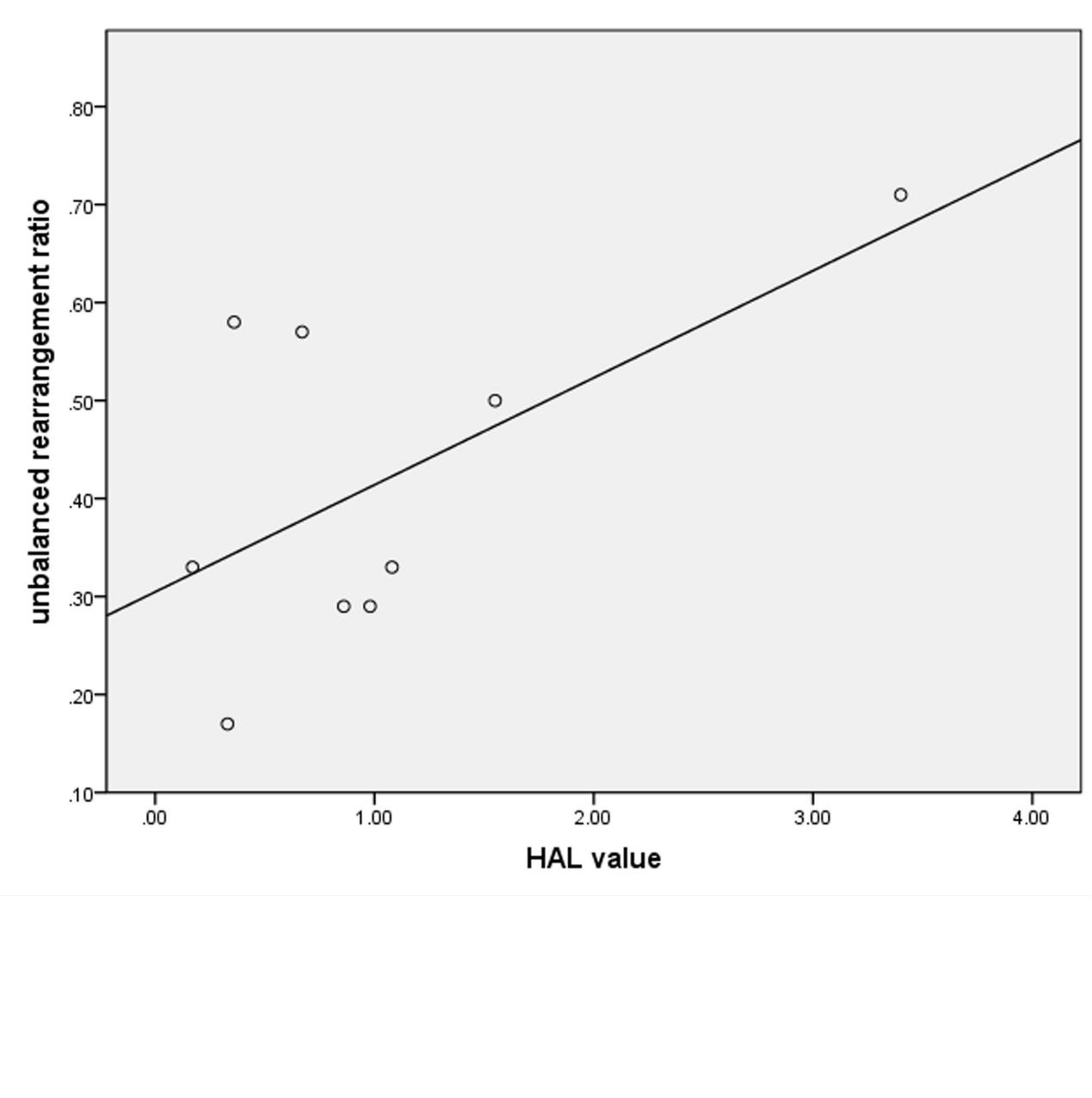


Supplementary Figure 2: Linear plot of autosomal haploid length (HAL) and unbalanced rearrangement rate, r=0.606，*P*=0.084.


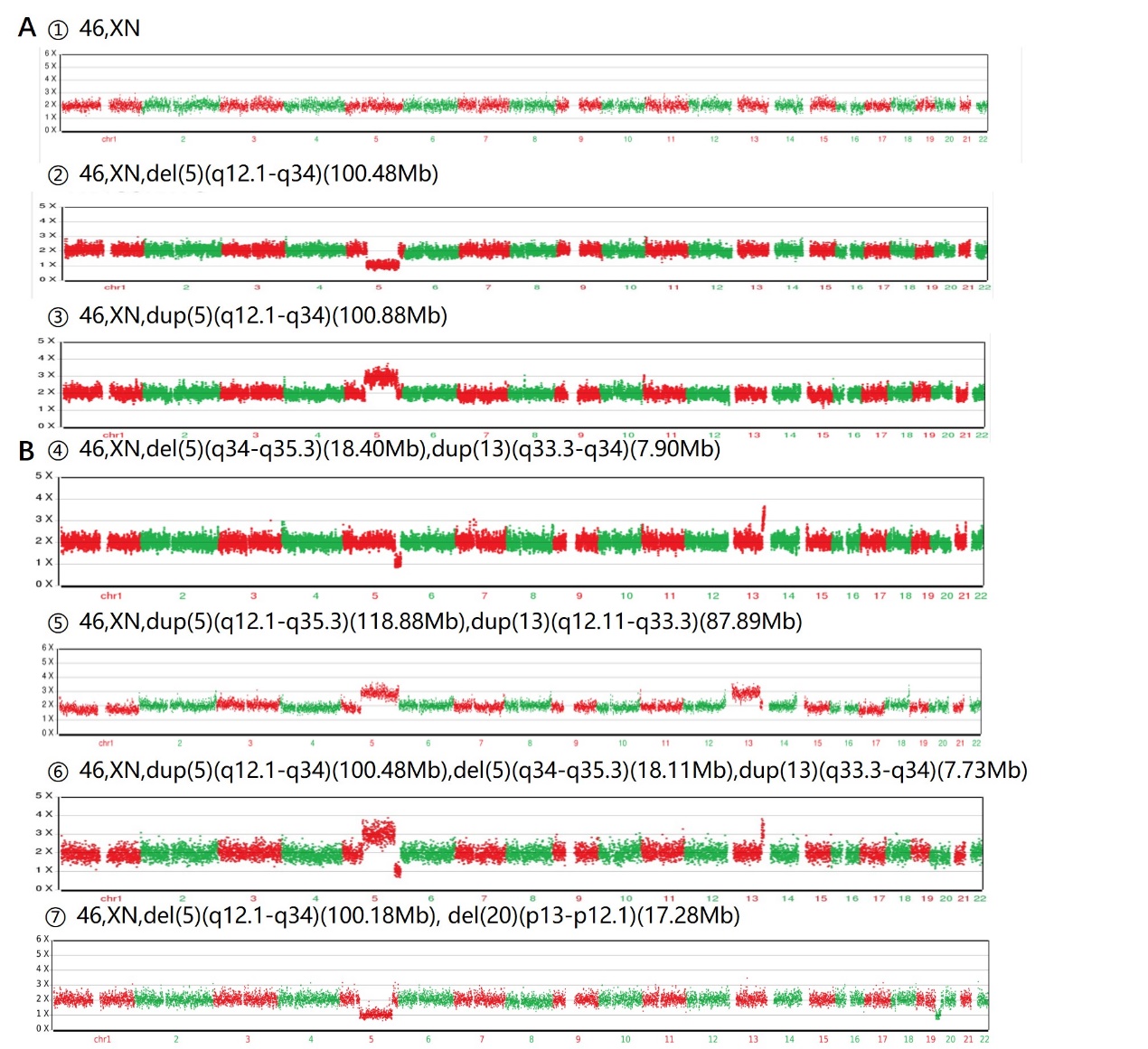


Supplementary Figure 3: CNVs result of Case15. A: the result of an embryo without crossover occurs during gametogenesis; B: the result of an embryo with crossover occurs during gametogenesis.

**References**

1. Gray JE, Syrett JE, Ritchie KM, Elliott WD. An interstitial translocation: chromosome no. 1p to 4q. Lancet (London, England) 1972;2:92-3.

2. Grace E, Sutherland GR, Bain AD. Familial insertional translocation. Lancet (London, England) 1972;2:231.

3. Rethoré MO, Lejeune J, Carpentier S, Prieur M, Dutrillaux B, Seringe P *et al.* [Trisomy for the distal part of the short arm of the number 3 chromosome in 3 siblings. First example of chromosomal insertion: INS(7;3)(q 31;p 21 p 26)]. Annales de genetique 1972;15:159-65.

4. Shapiro LR, Warburton D. Interstitial translocation in man. Lancet (London, England) 1972;2:712-3.

5. Berger R, Touati G, Derre J, Ortiz MA, Martinetti J. "Cri du chat" syndrome with maternal insertional translocation. Clinical genetics 1974;5:428-32.

6. Chudley AE, Bauder F, Ray M, McAlpine PJ, Pena SD, Hamerton JL. Familial mental retardation in a family with an inherited chromosome rearrangement. Journal of medical genetics 1974;11:353-66.

7. Lur'e IV, Gurevich DB, Laziuk GI. [Genetic counseling in cases of chromosome insertions]. Genetika 1975;11:131-6.

8. Pan SF, Fatora SR, Sorg R, Garver KL, Steele MW. Meiotic consequences of an intrachromosomal insertion of chromosome No 1: a family pedigree. Clinical genetics 1977;12:303-13.

9. George DL, Francke U. Regional mapping of human genes for hexosaminidase B and diphtheria toxin sensitivity on chromosome 5 using mouse X human hybrid cells. Somatic cell genetics 1977;3:629-38.

10. Back E, Kosmützky J, Schuwald A, Hameister H. Two cases of partial trisomy 10q in the same family caused by parental direct insertion [ins. (15;10) (q15;q24q26)]. Annales de genetique 1979;22:195-8.

11. Miller M, Kaufman G, Reed G, Bilenker R, Schinzel A. Familial, balanced insertional translocation of chromosome 7 leading to offspring with deletion and duplication of the inserted segment, 7p15 leads to 7p21. American journal of medical genetics 1979;4:323-32.

12. Riccardi VM, Hittner HM, Francke U, Pippin S, Holmquist GP, Kretzer FL *et al.* Partial triplication and deletion of 13q: study of a family presenting with bilateral retinoblastomas. Clinical genetics 1979;15:332-45.

13. Mattei MG, Mattei JF, Bernard R, Giraud F. Partial trisomy 4 resulting from a complex maternal rearrangement of chromosomes 2, 4, and 18 with interstitial translocation. Human genetics 1979;51:55-61.

14. Emanuel BS, Zackai EH, Moreau L, Coates P, Orrechio E. Interstitial deletion 13q33 resulting from maternal insertional translocation. Clinical genetics 1979;16:340-6.

15. Nielsen KB, Egede F, Mouridsen I, Mohr J. Familial partial 7q monosomy resulting from segregation of an insertional chromosome rearrangement. Journal of medical genetics 1979;16:461-6.

16. Beltran G, Varela M. Clonal changes in chronic granulocytic leukemia in blastic transformation and during remission. Cancer 1980;46:1590-3.

17. Williamson RA, Donlan MA, Dolan CR, Thuline HC, Harrison MT, Hall JG. Familial insertional translocation of a portion of 3q into 11q resulting in duplication and deletion of region 3q22.1 leads to q24 in different offspring. American journal of medical genetics 1981;9:105-11.

18. Strong LC, Riccardi VM, Ferrell RE, Sparkes RS. Familial retinoblastoma and chromosome 13 deletion transmitted via an insertional translocation. Science (New York, NY) 1981;213:1501-3.

19. Lessard M, Duval S, Fritz A. Unusual translocation and chronic myelocytic leukemia: "masked" Philadelphia chromosome (Ph 1). Cancer genetics and cytogenetics 1981;4:237-44.

20. Gilgenkrantz S, Dulucq P, Bresson JL, Gouget A, Pernot C, Gregoire MJ. Partial proximal trisomy of the long arm of chromosome 5 (q13 leads to q22) resulting from maternal insertion der ins (10;5). Journal of medical genetics 1981;18:465-9.

21. Pai GS, Rogers JF, Sommer A. Identical multiple congenital anomalies/mental retardation (MCA/MR) syndrome due to del(2)(q32) in two sisters with intrachromosomal insertional translocation in their father. American journal of medical genetics 1983;14:189-95.

22. Abe R, Ryan D, Cecalupo A, Cohen H, Sandberg AA. Cytogenetic findings in congenital leukemia: case report and review of the literature. Cancer genetics and cytogenetics 1983;9:139-44.

23. Kleczkowska A, Fryns JP, Van den Berghe H. Partial trisomy of chromosome 3(p14----p22) due to maternal insertional translocation. Annales de genetique 1984;27:180-3.

24. Orye E, Laureys G. Trisomy 3p syndrome. Report of a new case, due to a chromosomal insertion. Annales de genetique 1984;27:245-7.

25. Fryns JP, Kleczkowska A, Kenis H. De novo complex chromosomal rearrangement (CCR) in a severely mentally retarded boy. Annales de genetique 1984;27:62-4.

26. Fryns JP, Kleczkowska A, Lebas E, Goffaux P, Van den Berghe H. Complex chromosomal rearrangement in a mentally retarded boy without gross dysmorphic stigmata. Acta paediatrica Scandinavica 1984;73:138-40.

27. van de Vooren MJ, Planteydt HT, Hagemeijer A, Peters-Slough MF, Timmerman MJ. Familial balanced insertion (5;10) and monosomy and trisomy (10) (q24.2----q25.3). Clinical genetics 1984;25:52-8.

28. Watt JL, Couzin DA, Lloyd DJ, Stephen GS, McKay E. A familial insertion involving an active nucleolar organiser within chromosome 12. Journal of medical genetics 1984;21:379-84.

29. Hecht F, Morgan R, Schrier SL, Adams J, Sandberg AA. The Philadelphia (Ph) chromosome in leukemia. I. A new mechanism due to interstitial deletion and insertion in chronic myelocytic leukemia. Cancer genetics and cytogenetics 1985;14:3-10.

30. Daeschner C, 3rd, Elder F, Carpentieri U, Haggard ME. Leukemia with a novel 4q11q rearrangement. Cancer genetics and cytogenetics 1985;16:245-50.

31. Turchini MF, Travade P, Geneix A, De Laroque A, Bezou MJ, Malet P. Insertion of part of chromosome 5 into chromosome 1 in a case of sideroblastic anemia with an excess of blasts. Cancer genetics and cytogenetics 1985;16:353-5.

32. Delozier-Blanchet CD, Pitmon D, Schorderet D, Engel E. [Cri-du-chat syndrome and two other deformed children in a family carrying a pericentric inversion or insertion of chromosome 5]. Journal de genetique humaine 1985;33:371-80.

33. Rege-Cambrin G, Mecucci C, Van Orshoven A, Tricot G, van den Berghe H. 11q23 involvement in a complex insertion (10; 11) in acute monocytic leukemia. Leukemia research 1986;10:1159-61.

34. Walker AP, Bocian M. Partial duplication 8q12----q21.2 in two sibs with maternally derived insertional and reciprocal translocations: case reports and review of partial duplications of chromosome 8. American journal of medical genetics 1987;27:3-22.

35. Donti E, Falini B, Bordoni S, Rosetti A, Carloni I, Donti GV. ins(6;11) in a case of peripheral T-cell lymphoma. Cancer genetics and cytogenetics 1987;27:367-9.

36. Ueda T, Aozasa K, Tsujimoto M, Uchida A, Taniwaki M, Abe T. Translocation X;18 and insertion 15;11 in a case of synovial sarcoma. Cancer genetics and cytogenetics 1988;30:183-5.

37. Forsythe MG, Walker H, Weiss L, Roberson JR, Worsham MJ, Babu VR *et al.* Duplication and deletion 11q23-q24 recombinants in two offspring of an intrachromosomal insertion ("shift") carrier. Henry Ford Hospital medical journal 1988;36:183-6.

38. Gustavson KH, Lundberg PO, Nicol P. Familial partial trisomy 5p resulting from segregation of an insertional translocation. Clinical genetics 1988;33:404-9.

39. Mugneret F, Lizard-Nacol S, Volk C, Cuisenier J, Colin F, Turc-Carel C. Association of breakpoint 14q23 with uterine leiomyoma. Cancer genetics and cytogenetics 1988;34:201-6.

40. Abuelo DN, Barsel-Bowers G, Richardson A. Insertional translocations: report of two new families and review of the literature. American journal of medical genetics 1988;31:319-29.

41. Lavedan C, Barichard F, Azoulay M, Couillin P, Molina Gomez D, Nicolas H *et al.* Molecular definition of de novo and genetically transmitted WAGR-associated rearrangements of 11p13. Cytogenetics and cell genetics 1989;50:70-4.

42. Yip MY, Kemp J, Hanson N, Wilson M, Purvis-Smith S, Lam-Po-Tang PR. Duplication of 5q11.2----q13.1 from a familial (5;20) balanced insertion. American journal of medical genetics 1989;33:220-3.

43. Edelhoff S, Maier B, Trautmann U, Pfeiffer RA. Interstitial deletion of 16(q13q22) in a newborn resulting from a paternal insertional translocation. Annales de genetique 1991;34:85-9.

44. Till M, Devillard F, Crost P, Bachy M, Prieur F, Berthéas MF. Balanced complex chromosomal rearrangements with more than four breakpoints: report of a new case. American journal of medical genetics 1991;40:370-3.

45. Muneer RS, Thompson LM, Kamat E. A rare insertional translocation of proximal segment with heterochromatic region of 1q into 7p in monozygotic twins and spontaneous abortions. Human genetics 1991;88:122-3.

46. Tümer Z, Tommerup N, Tønnesen T, Kreuder J, Craig IW, Horn N. Mapping of the Menkes locus to Xq13.3 distal to the X-inactivation center by an intrachromosomal insertion of the segment Xq13.3-q21.2. Human genetics 1992;88:668-72.

47. Goldman AS, Martin RH, Johannisson R, Gould CP, Davison EV, Emslie JE *et al.* Meiotic and sperm chromosome analysis in a male carrier of an inverted insertion (3;10)(q13.2;p14p13). Journal of medical genetics 1992;29:460-4.

48. Stengel-Rutkowski S, Lohse K, Herzog C, Apacik C, Couturier J, Albert A *et al.* Partial trisomy 8q. Two case reports with maternal translocation and inverted insertion: phenotype analyses and reflections on the risk. Clinical genetics 1992;42:178-85.

49. Shaffer LG, Hecht JT, Ledbetter DH, Greenberg F. Familial interstitial deletion 11(p11.12p12) associated with parietal foramina, brachymicrocephaly, and mental retardation. American journal of medical genetics 1993;45:581-3.

50. Naritomi K, Izumikawa Y, Tohma T, Hirayama K. Inverted insertion of chromosome 7q and ectrodactyly. American journal of medical genetics 1993;46:492-3.

51. Kobayashi H, Espinosa R, 3rd, Thirman MJ, Gill HJ, Fernald AA, Diaz MO *et al.* Heterogeneity of breakpoints of 11q23 rearrangements in hematologic malignancies identified with fluorescence in situ hybridization. Blood 1993;82:547-51.

52. Batista DA, Pai GS, Stetten G. Molecular analysis of a complex chromosomal rearrangement and a review of familial cases. American journal of medical genetics 1994;53:255-63.

53. Wagstaff J, Hemann M. A familial "balanced" 3;9 translocation with cryptic 8q insertion leading to deletion and duplication of 9p23 loci in siblings. American journal of human genetics 1995;56:302-9.

54. al-Attia HM, Sedaghatian MR. Mental retardation/shortness of stature/multiple minor anomalies syndrome associated with insertion of 3q material into 18p. American journal of medical genetics 1995;56:35-8.

55. Röijer E, Kas K, Van de Ven W, Stenman G. Mapping of the 8q12 translocation breakpoint to a 40-kb region in a pleomorphic adenoma with an ins(8;3)(q12;p21.3p14.1). Cytogenetics and cell genetics 1997;76:23-6.

56. Doheny KF, Rasmussen SA, Rutberg J, Semenza GL, Stamberg J, Schwartz M *et al.* Segregation of a familial balanced (12;10) insertion resulting in Dup(10)(q21.2q22.1) and Del(10)(q21.2q22.1) in first cousins. American journal of medical genetics 1997;69:188-93.

57. Senger G, Chudoba I, Friedrich U, Tommerup N, Claussen U, Brøndum-Nielsen K. Prenatal diagnosis of a half-cryptic translocation using chromosome microdissection. Prenatal diagnosis 1997;17:369-74.

58. Mrózek K, Szumigala J, Brooks JS, Crossland DM, Karakousis CP, Bloomfield CD. Round cell liposarcoma with the insertion (12;16)(q13;p11.2p13). American journal of clinical pathology 1997;108:35-9.

59. Nordgren A, Arver S, Kvist U, Carter N, Blennow E. Trisomy 5q12-->q13.3 in a patient with add(13q): characterization of an interchromosomal insertion by forward and reverse chromosome painting. American journal of medical genetics 1997;73:351-5.

60. Asamoah A, Nandi KN, Prouty L, Thurmon TF, Chen H. A case of insertional translocation involving chromosomes 2 and 4. Clinical genetics 1998;53:142-6.

61. Lessard M, Herry A, Berthou C, Léglise MC, Abgrall JF, Morice P *et al.* FISH investigation of 5q and 7q deletions in MDS/AML reveals hidden translocations, insertions and fragmentations of the same chromosomes. Leukemia research 1998;22:303-12.

62. Park JP, Moeschler JB, Davies WS, Patel PI, Mohandas TK. Smith-Magenis syndrome resulting from a de novo direct insertion of proximal 17q into 17p11.2. American journal of medical genetics 1998;77:23-7.

63. Martineau M, Berger R, Lillington DM, Moorman AV, Secker-Walker LM. The t(6;11)(q27;q23) translocation in acute leukemia: a laboratory and clinical study of 30 cases. EU Concerted Action 11q23 Workshop participants. Leukemia 1998;12:788-91.

64. Vust A, Riordan D, Wickstrom D, Chudley AE, Dawson AJ. Functional mosaic trisomy of 1q12-->1q21 resulting from X-autosome insertion translocation with random inactivation. Clinical genetics 1998;54:70-3.

65. Lukusa T, Fryns JP. Syndrome of facial, oral, and digital anomalies due to 7q21.2-->q22.1 duplication. American journal of medical genetics 1998;80:454-8.

66. Utkus A, Sorokina I, Kucinskas V, Röthlisberger B, Balmer D, Brecevic L *et al.* Duplication of segment 1p21 following paternal insertional translocation, ins(6;1)(q25;p13.3p22.1). Journal of medical genetics 1999;36:73-6.

67. Ramos PS, Bitgood JJ, Ponce de León FA. Novel chromosomal insertional translocation in chicken uncovered by double color FISH. Animal biotechnology 1999;10:119-22.

68. Fan YS, Rizkalla K, Barr RM. A new complex variant Philadelphia chromosome, t(1;9;22)ins(17;22), characterized by fluorescence in situ hybridization in an adult ALL. Leukemia research 1999;23:1001-6.

69. Fritz B, Müller-Navia J, Hillig U, Köhler M, Aslan M, Rehder H. Trisomy 2q35-q37 due to insertion of 2q material into 17q25: clinical, cytogenetic, and molecular cytogenetic characterization. American journal of medical genetics 1999;87:297-301.

70. Kokalj-Vokac N, Medica I, Zagorac A, Zagradisnik B, Erjavec A, Gregoric A. A case of insertional translocation resulting in partial trisomy 16p. Annales de genetique 2000;43:131-5.

71. Van Hemel JO, Eussen HJ. Interchromosomal insertions. Identification of five cases and a review. Human genetics 2000;107:415-32.

72. Kaiser-Rogers KA, Rao KW, Michaelis RC, Lese CM, Powell CM. Usefulness and limitations of FISH to characterize partially cryptic complex chromosome rearrangements. American journal of medical genetics 2000;95:28-35.

73. Cai T, Yu P, Tagle DA, Lu D, Chen Y, Xia J. A de novo complex chromosomal rearrangement with a translocation 7;9 and 8q insertion in a male carrier with no infertility. Human reproduction (Oxford, England) 2001;16:59-62.

74. Martín-Subero JI, Lahortiga I, Gómez E, Ferreira C, Larrayoz MJ, Odero MD *et al.* Insertion (22;9)(q11;q34q21) in a patient with chronic myeloid leukemia characterized by fluorescence in situ hybridization. Cancer genetics and cytogenetics 2001;125:167-70.

75. Zhao L, Hayes K, Khan Z, Glassman A. Spectral karyotyping study of chromosome abnormalities in human leukemia. Cancer genetics and cytogenetics 2001;127:143-7.

76. Elias WJ, Lopes MB, Golden WL, Jane JA, Sr., Gonzalez-Fernandez F. Trilateral retinoblastoma variant indicative of the relevance of the retinoblastoma tumor-suppressor pathway to medulloblastomas in humans. Journal of neurosurgery 2001;95:871-8.

77. Eisenhut M, Weindling M, Fryer A. Amyoplasia associated with a balanced de novo insertion of a segment of the long arm of chromosome 5 into chromosome 2. American journal of medical genetics 2002;107:337-9.

78. Rolston R, Weck KE, Tersak JM, Sherer ME, Cumbie K, Shekhter-Levin S. New cytogenetic variant, insertion (15;17)(q22;q12q21), in an adolescent with acute promyelocytic leukemia. Cancer genetics and cytogenetics 2002;134:55-9.

79. Tan Y, Lu G. Chromosomal cryptic insertion of the terminal region and its formative mechanism determined by fluorescence in situ hybridization. Chinese medical journal 2002;115:1039-42.

80. Watanabe N, Kobayashi H, Hirama T, Kikuta A, Koizumi S, Tsuru T *et al.* Cryptic t(12;15)(p13;q26) producing the ETV6-NTRK3 fusion gene and no loss of IGF2 imprinting in congenital mesoblastic nephroma with trisomy 11: fluorescence in situ hybridization and IGF2 allelic expression analysis. Cancer genetics and cytogenetics 2002;136:10-6.

81. de Pater JM, Ippel PF, van Dam WM, Loneus WH, Engelen JJ. Characterization of partial trisomy 9p due to insertional translocation by chromosomal (micro)FISH. Clinical genetics 2002;62:482-7.

82. Matteucci C, La Starza R, Crescenzi B, Romoli S, Santoro A, Magrin S *et al.* Different mechanisms lead to a karyotypically identical t(20;21) in myelodysplastic syndrome and in acute myelocytic leukemia. Cancer genetics and cytogenetics 2003;140:13-7.

83. Domínguez MG, Wong-Ley LE, Rivera H, Vásquez AI, Ramos AL, Sánchez-Urbina R *et al.* Pure partial trisomy 6p due to a familial insertion (16;6)(p12;p21.2p23). Annales de genetique 2003;46:45-8.

84. Grasshoff U, Singer S, Liehr T, Starke H, Fode B, Schöning M *et al.* A complex chromosomal rearrangement with a translocation 4;10;14 in a fertile male carrier: ascertainment through an offspring with partial trisomy 14q13-->q24.1 and partial monosomy 4q27-->q28 [corrected]. Cytogenetic and genome research 2003;103:17-23.

85. Martin DM, Mindell MH, Kwierant CA, Glover TW, Gorski JL. Interrupted aortic arch in a child with trisomy 5q31.1q35.1 due to a maternal (20;5) balanced insertion. American journal of medical genetics Part A 2003;116a:268-71.

86. Douet-Guilbert N, Morel F, Le Bris MJ, Herry A, Le Calvez G, Marion V *et al.* Translocation (12;21) followed by insertion of chromosome 3 material in the derivative chromosome 12 in a case of childhood acute lymphoblastic leukemia. Cancer genetics and cytogenetics 2003;142:120-3.

87. Engelen JJ, Arens YH, Gondrie ET, Alofs MG, Loneus WH, Hamers AJ. Intrachromosomal insertion translocation resulting in duplication of chromosome band Yq11.2 in two fertile brothers. American journal of medical genetics Part A 2003;118a:287-9.

88. Punnett A, Teshima I, Heon E, Budning A, Sutherland J, Gallie BL *et al.* Unique insertional translocation in a childhood Wilms' tumor survivor detected when his daughter developed bilateral retinoblastoma. American journal of medical genetics Part A 2003;120a:105-9.

89. Birch NC, Antonescu CR, Nelson M, Sarran L, Neff JR, Seemayer T *et al.* Inconspicuous insertion 22;12 in myxoid/round cell liposarcoma accompanied by the secondary structural abnormality der(16)t(1;16). The Journal of molecular diagnostics : JMD 2003;5:191-4.

90. Morel F, Le Bris MJ, Douet-Guilbert N, Duchemin J, Herry A, Le Calvez G *et al.* Insertion of chromosome 11 in chromosome 4 resulting in a 5'MLL-3'AF4 fusion gene in a case of adult acute lymphoblastic leukemia. Cancer genetics and cytogenetics 2003;145:74-7.

91. Onozawa M, Fukuhara T, Nigo M, Takeda A, Takahata M, Yamamoto Y *et al.* Insertion (21;8)(q22;q22q22): a masked t(8;21) in a patient with acute myelocytic leukemia. Cancer genetics and cytogenetics 2003;147:134-9.

92. Chen SN, Xue YQ, Wu YF, Pan JL. [Cytogenetic and molecular genetic studies on a variant of t(15;17), ins(17;15)(q21;q14q22), in an acute promyelocytic leukemia patient]. Zhonghua yi xue yi chuan xue za zhi = Zhonghua yixue yichuanxue zazhi = Chinese journal of medical genetics 2004;21:77-9.

93. Lafay-Cousin L, Soenen V, Mazingue F, Preudhomme C, Laï JL, Andrieux J. Chromosomal insertion involving MLL in childhood acute myeloblastic leukemia (M4). Cancer genetics and cytogenetics 2004;150:153-5.

94. Shim SH, Wyandt HE, McDonald-McGinn DM, Zackai EZ, Milunsky A. Molecular cytogenetic characterization of multiple intrachromosomal rearrangements of chromosome 2q in a patient with Waardenburg's syndrome and other congenital defects. Clinical genetics 2004;66:46-52.

95. Vermeesch JR, Thoelen R, Fryns JP. A familial complex chromosome translocation resulting in duplication of 6p25. Annales de genetique 2004;47:275-80.

96. Lespinasse J, Réthoré MO, North MO, Bovier-Lapierre M, Lundsteen C, Fert-Ferrer S *et al.* Balanced complex chromosomal rearrangements (BCCR) with at least three chromosomes and three or more breakpoints: report of three new cases. Annales de genetique 2004;47:315-24.

97. Arnaud B, Morel F, Douet-Guilbert N, Le Bris MJ, De Braekeleer M. X chromosome insertion in the MLL gene in a case of childhood acute myeloblastic leukemia. Cancer genetics and cytogenetics 2004;152:149-52.

98. Arens YH, Engelen JJ, Govaerts LC, van Ravenswaay CM, Loneus WH, van Lent-Albrechts JC *et al.* Familial insertion (3;5)(q25.3;q22.1q31.3) with deletion or duplication of chromosome region 5q22.1-5q31.3 in ten unbalanced carriers. American journal of medical genetics Part A 2004;130a:128-33.

99. Melotte C, Debrock S, D'Hooghe T, Fryns JP, Vermeesch JR. Preimplantation genetic diagnosis for an insertional translocation carrier. Human reproduction (Oxford, England) 2004;19:2777-83.

100. Han JY, Kim KH, Jun HJ, Je GH, Glotzbach CD, Shaffer LG. Partial trisomy of chromosome 10(q22-q24) due to maternal insertional translocation (15;10). American journal of medical genetics Part A 2004;131:190-3.

101. Lee J, Hopcus-Niccum DJ, Mulvihill JJ, Li S. Cytogenetic and molecular cytogenetic studies of a variant of t(21;22), ins(22;21)(q12;q21q22), with a deletion of the 3' EWSR1 gene in a patient with Ewing sarcoma. Cancer genetics and cytogenetics 2005;159:177-80.

102. de Ravel T, Aerssens P, Vermeesch JR, Fryns JP. Trisomy of chromosome 16p13.3 due to an unbalanced insertional translocation into chromosome 22p13. European journal of medical genetics 2005;48:355-9.

103. Surace C, Storlazzi CT, Engellau J, Domanski HA, Gustafson P, Panagopoulos I *et al.* Molecular cytogenetic characterization of an ins(4;X) occurring as the sole abnormality in an aggressive, poorly differentiated soft tissue sarcoma. Virchows Archiv : an international journal of pathology 2005;447:869-74.

104. Malmgren H, Malm G, Sahlén S, Karlsson M, Blennow E. Molecular cytogenetic characterization of an insertional translocation, ins(6;7)(p25;q33q34): deletion/duplication of 7q33-34 and clinical correlations. American journal of medical genetics Part A 2005;139:25-31.

105. Tirado CA, Meloni-Ehrig AM, Edwards T, Scheerle J, Burks K, Repetti C *et al.* Cryptic ins(4;11)(q21;q23q23) detected by fluorescence in situ hybridization: a variant of t(4;11)(q21;q23) in an infant with a precursor B-cell acute lymphoblastic leukemia report of a second case. Cancer genetics and cytogenetics 2007;174:166-9.

106. de Oliveira FM, Falcão RP, de Figueiredo Pontes LL, Simões BP, Tone LG. Insertion (15;14)(q22;q13q32) in a case of Ph+ ALL. Cancer genetics and cytogenetics 2008;185:65-7.

107. Schulz S, Volleth M, Muschke P, Wieland I, Wieacker P. Greig cephalopolysyndactyly (GCPS) contiguous gene syndrome in a boy with a 14 Mb deletion in region 7p13-14 caused by a paternal balanced insertion (5; 7). The application of clinical genetics 2008;1:19-22.

108. Bai S, Xue Y, Wu Y, Pan J, Zhang J, Shen J *et al.* [Simultaneous presence of ins (15;17),t(2;17;20) and trisomy 8 in a patient with acute promyelocytic leukemia]. Zhonghua yi xue yi chuan xue za zhi = Zhonghua yixue yichuanxue zazhi = Chinese journal of medical genetics 2008;25:712-4.

109. Luo YQ, Shen M, Qian YL, Chen YL, Xu CM, Jin F. [Genetic analysis of a complex chromosome rearrangement involving two chromosomes and four breakpoints in an azoospermic man]. Zhonghua yi xue yi chuan xue za zhi = Zhonghua yixue yichuanxue zazhi = Chinese journal of medical genetics 2009;26:200-2.

110. Tzschach A, Ramel C, Kron A, Seipel B, Wüster C, Cordes U *et al.* Hypergonadotropic hypogonadism in a patient with inv ins (2;4). International journal of andrology 2009;32:226-30.

111. Van Zutven LJ, van Bever Y, Van Nieuwland CC, Huijbregts GC, Van Opstal D, von Bergh AR *et al.* Interstitial 11q deletion derived from a maternal ins(4;11)(p14;q24.2q25): a patient report and review. American journal of medical genetics Part A 2009;149a:1468-75.

112. Gruchy N, Barreau M, Kessler K, Gourdier D, Leporrier N. A paternally transmitted complex chromosomal rearrangement (CCR) involving chromosomes 2, 6, and 18 includes eight breakpoints and five insertional translocations (ITs) through three generations. American journal of medical genetics Part A 2010;152a:185-90.

113. Jang JH, Yoo EH, Kim HJ, Kim DH, Jung CW, Kim SH. Acute myeloid leukemia with del(X)(p21) and cryptic RUNX1/RUNX1T1 from ins(8;21)(q22;q22q22) revealed by atypical FISH signals. Annals of clinical and laboratory science 2010;40:80-4.

114. Shearer BM, Sukov WR, Flynn HC, Knudson RA, Ketterling RP. Development of a dual-color, double fusion FISH assay to detect RPN1/EVI1 gene fusion associated with inv(3), t(3;3), and ins(3;3) in patients with myelodysplasia and acute myeloid leukemia. American journal of hematology 2010;85:569-74.

115. de Figueiredo AF, Liehr T, Bath S, Binato R, Ventura EM, de Souza MT *et al.* A new cryptic ins(11;1)(q23;q21q31) detected in a t(1;8;11)(q21;p21;q23) in a baby with acute myeloid leukemia FAB AML-M5. Blood cells, molecules & diseases 2010;45:197-8.

116. Spreiz A, Müller D, Zotter S, Albrecht U, Baumann M, Fauth C *et al.* Phenotypic variability of a deletion and duplication 6q16.1 → q21 due to a paternal balanced ins(7;6)(p15;q16.1q21). American journal of medical genetics Part A 2010;152a:2762-7.

117. Rücker FG, Bullinger L, Gribov A, Sill M, Schlenk RF, Lichter P *et al.* Molecular characterization of AML with ins(21;8)(q22;q22q22) reveals similarity to t(8;21) AML. Genes, chromosomes & cancer 2011;50:51-8.

118. Vanneste E, Melotte C, Voet T, Robberecht C, Debrock S, Pexsters A *et al.* PGD for a complex chromosomal rearrangement by array comparative genomic hybridization. Human reproduction (Oxford, England) 2011;26:941-9.

119. Xiao B, Zhang JM, Ji X, Jiang WT, Hu J, Tao J. [Two cases of partial trisomy 8p derived from paternal reciprocal translocation or maternal insertion translocation: clinical features and genetic abnormalities]. Zhonghua yi xue yi chuan xue za zhi = Zhonghua yixue yichuanxue zazhi = Chinese journal of medical genetics 2011;28:247-50.

120. Singh S, Ashton F, Marquis-Nicholson R, Love JM, Lan CC, Aftimos S *et al.* A novel 2.3 mb microduplication of 9q34.3 inserted into 19q13.4 in a patient with learning disabilities. Case reports in pediatrics 2012;2012:459602.

121. Ashaat N, Husseiny A. Correlation between missed abortion and insertional translocation involving chromosomes 1 and 7. Iranian journal of reproductive medicine 2012;10:15-22.

122. Assawamakin A, Wattanasirichaigoon D, Tocharoentanaphol C, Waeteekul S, Tansatit M, Thongnoppakhun W *et al.* A novel maternally-derived insertional translocation resulting in partial trisomy 4q13.2-q22.1 with complex translocation t(8;20) in a family with intellectual disability. American journal of medical genetics Part A 2012;158a:901-8.

123. Quinonez SC, Hedera P, Barr M, Ackley T, Lam C, Purkayastha A *et al.* Maternal intrachromosomal insertional translocation leads to recurrent 1q21.3q23.3 deletion in two siblings. American journal of medical genetics Part A 2012;158a:2591-601.

124. Simioni M, Vieira TP, Sgardioli IC, Freitas EL, Rosenberg C, Maurer-Morelli CV *et al.* Insertional translocation of 15q25-q26 into 11p13 and duplication at 8p23.1 characterized by high resolution arrays in a boy with congenital malformations and aniridia. American journal of medical genetics Part A 2012;158a:2905-10.

125. Boles J, DeNicola M, Collins R, Garcia R, Patel S, Satayasoontorn K *et al.* A Chronic Myelogenous Leukemia (CML) Case with a Cryptic Insertion of the ABL1 Gene of Chromosome 9 into 22 Resulting in a Fusion Signal on the Derivative 22: 46,XY.ish ins(22;9)(q11.2;q34q34)BCR+,ABL1. Journal of the Association of Genetic Technologists 2013;39:21-2.

126. Matoso E, Melo JB, Ferreira SI, Jardim A, Castelo TM, Weise A *et al.* Insertional translocation leading to a 4q13 duplication including the EPHA5 gene in two siblings with attention-deficit hyperactivity disorder. American journal of medical genetics Part A 2013;161a:1923-8.

127. Min BJ, Ko JM, Seo ME, Choi JS, Oh SK, Jeon J *et al.* An interstitial, apparently-balanced chromosomal insertion in the etiology of Langer-Giedion syndrome in an Asian family. European journal of medical genetics 2013;56:561-5.

128. Topcu V, Ilgin-Ruhi H, Yurur-Kutlay N, Ekici C, Vicdan A, Tukun FA. Pure partial trisomy 4q syndrome in a child with der(9)ins(9;4)(q34.3;q26q35.2)mat. Genetic counseling (Geneva, Switzerland) 2014;25:1-6.

129. Jarošová M, Rohoň P, Živná J, Peková S, Nedomová R, Holzerová M *et al.* Pathogenetic role of ETV6 fusion gene in leukemic transformation of myelodysplastic syndrome refractory anemia with excess blasts-1 with a new, rare translocation t(11;19)(q24.3;q13.12) and insertion ins(6;12)(p22.3p13). Leukemia & lymphoma 2014;55:950-3.

130. Deng L, Peng Y, Liu J, Wen J, Xia Y, Liang D *et al.* Brief report. Adult patient presenting an interstitial (9) (q21.32q31.1) direct duplication resulting from the malsegregation of a paternal balanced insertional translocation. Birth defects research Part A, Clinical and molecular teratology 2014;100:294-9.

131. Li L, Chen H, Yin C, Yang C, Wang B, Zheng S *et al.* Mapping breakpoints of a familial chromosome insertion (18,7) (q22.1; q36.2q21.11) to DPP6 and CACNA2D1 genes in an azoospermic male. Gene 2014;547:43-9.

132. Kjeldsen E. A novel insertion ins(18;5)(q21.1;q31.2q35.1) in acute myeloid leukemia associated with microdeletions at 5q31.2, 5q35.1q35.2 and 18q12.3q21.1 detected by oligobased array comparative genomic hybridization. Molecular cytogenetics 2014;7:63.

133. Matveeva E, Kazakova A, Olshanskaya Y, Tsaur G, Shelikhova L, Meyer C *et al.* A new variant of KMT2A(MLL)-FLNA fusion transcript in acute myeloid leukemia with ins(X;11)(q28;q23q23). Cancer genetics 2015;208:148-51.

134. Guan H, Liu J, Guo X, Wu C, Yu H. Microgranular variant of acute promyelocytic leukemia with der(17) ins(17;15): A case report and review of the literature. Experimental and therapeutic medicine 2015;10:1009-12.

135. Wang Z, Zen W, Meng F, Xin X, Luo L, Sun H *et al.* Chronic myeloid leukemia with variation of translocation at (Ph) [ins (22;9) (q11;q21q34)]: a case report. International journal of clinical and experimental pathology 2015;8:13707-10.

136. Lentes J, Thomay K, Schneider DT, Bernbeck B, Reinhardt D, Marschalek R *et al.* Identification of a Cryptic Insertion ins(11;X)(q23;q28q12) Resulting in a KMT2A-FLNA Fusion in a 13-Month-Old Child with Acute Myelomonocytic Leukemia. Cytogenetic and genome research 2016;150:281-6.

137. Margolskee E, Saab J, Geyer JT, Aledo A, Mathew S. A Novel Variant t(1;22) Translocation - ins(22;1)(q13;p13p31) - in a Child with Acute Megakaryoblastic Leukemia. The American journal of case reports 2017;18:422-6.

138. Luo A, Cheng D, Yuan S, Li H, Du J, Zhang Y *et al.* Maternal interchromosomal insertional translocation leading to 1q43-q44 deletion and duplication in two siblings. Molecular cytogenetics 2018;11:24.

139. Sihombing NRB, de Leeuw N, van Bokhoven H, Faradz SM. Duplication of 1q31.3q41 in two affected siblings due to paternal insertional translocation. BMJ case reports 2019;12.

140. Dong Z, Chau MHK, Zhang Y, Dai P, Zhu X, Leung TY *et al.* Deciphering the complexity of simple chromosomal insertions by genome sequencing. Human genetics 2021;140:361-80.

141. Zenagui R, Bernicot I, Ranisavljevic N, Ferrieres-Hoa A, Puechberty J, Anahory T. Whole-genome analysis of a putative rare and complex interchromosomal reciprocal insertion: thorough investigations for a straightforward interpretation. Reproductive biomedicine online 2022;44:636-40.

142. Berg HE, Greipp PT, Baughn LB, Falcon CP, Jackson CC, Peterson JF. Detection of a Cryptic KMT2A/AFDN Gene Fusion [ins(6;11)(q27;q23q23)] in a Pediatric Patient with Newly Diagnosed Acute Myeloid Leukemia. Laboratory medicine 2022;53:e95-e9.
